# Visuomotor behaviours when using a myoelectric prosthesis

**DOI:** 10.1186/1743-0003-11-72

**Published:** 2014-04-23

**Authors:** Mohammad M D Sobuh, Laurence P J Kenney, Adam J Galpin, Sibylle B Thies, Jane McLaughlin, Jai Kulkarni, Peter Kyberd

**Affiliations:** 1 Department of Orthotics and Prosthetics, Faculty of Rehabilitation Sciences, University of Jordan, Amman, Jordan; 2 Centre for Health Sciences Research, University of Salford, Salford, UK; 3 School of Health Sciences, University of Salford, Salford, UK; 4 Manchester Disablement Services Centre, Withington Hospital, Manchester, UK; 5 Institute of Biomedical Engineering, University of New Brunswick, Fredericton, NB, Canada

**Keywords:** Prosthesis, Myoelectric, Visuomotor behaviour, Design evaluation, Amputee, Upper limb

## Abstract

**Background:**

A recent study showed that the gaze patterns of amputee users of myoelectric prostheses differ markedly from those seen in anatomically intact subjects. Gaze behaviour is a promising outcome measures for prosthesis designers, as it appears to reflect the strategies adopted by amputees to compensate for the absence of proprioceptive feedback and uncertainty/delays in the control system, factors believed to be central to the difficulty in using prostheses. The primary aim of our study was to characterise visuomotor behaviours over learning to use a trans-radial myoelectric prosthesis. Secondly, as there are logistical advantages to using anatomically intact subjects in prosthesis evaluation studies, we investigated similarities in visuomotor behaviours between anatomically intact users of a trans-radial prosthesis simulator and experienced trans-radial myoelectric prosthesis users.

**Methods:**

In part 1 of the study, we investigated visuomotor behaviours during performance of a functional task (reaching, grasping and manipulating a carton) in a group of seven anatomically intact subjects over learning to use a trans-radial myoelectric prosthesis simulator (Dataset 1). Secondly, we compared their patterns of visuomotor behaviour with those of four experienced trans-radial myoelectric prosthesis users (Dataset 2). We recorded task movement time, performance on the SHAP test of hand function and gaze behaviour.

**Results:**

Dataset 1 showed that while reaching and grasping the object, anatomically intact subjects using the prosthesis simulator devoted around 90% of their visual attention to either the hand or the area of the object to be grasped. This pattern of behaviour did not change with training, and similar patterns were seen in Dataset 2. Anatomically intact subjects exhibited significant increases in task duration at their first attempts to use the prosthesis simulator. At the end of training, the values had decreased and were similar to those seen in Dataset 2.

**Conclusions:**

The study provides the first functional description of the gaze behaviours seen during use of a myoelectric prosthesis. Gaze behaviours were found to be relatively insensitive to practice. In addition, encouraging similarities were seen between the amputee group and the prosthesis simulator group.

## Background

Trans-radial myoelectric prostheses are operated via electromyographic (EMG) signals measured at the residual forearm musculature. They differ markedly from the anatomic hand in a number of ways, including their mass properties, the greatly limited controllable degrees of freedom, and absence of proprioceptive feedback from the hand and wrist [1]. Hence, it is not surprising that such devices are challenging to use and often poorly utilized, or rejected [2,3]. Indeed, the difficulty in controlling a prosthesis has long been considered one of the limiting factors in the field of myoelectric prostheses and one of the key reasons cited by prosthesis rejecters [4].

Significant efforts are now being devoted to develop improved prosthesis control strategies with renewed interest in artificial proprioception [5], EMG pattern recognition [6] and hierarchical control [7,8], but the speed of development may be being limited by the evaluation tools available to the designers. For example, questionnaire and interview-based approaches to measure ease of use, and frequency of use in everyday life can only be used once a new prosthesis reaches a mature stage in its development. Clinical measures based on ordinal scales, although applicable to the evaluation of prototypes, are insensitive, reliant on the rater’s skills and hence poor substitutes for objective measurement tools. The most objective of the commonly used upper limb evaluation tools are based on time to perform a structured set of tasks (e.g. [9]), but use of these in isolation gives limited insight into the ease of use of a prosthesis. To further compound the difficulties faced by designers of novel upper limb prostheses, it is difficult to recruit large numbers of upper limb prosthesis users for clinical evaluation studies, leading a number of early stage design studies to focus on participants who are anatomically intact [10,11].

To identify more promising methods for evaluation of prostheses that could be used in early stage studies of novel designs, it is first necessary to better understand what factors are most closely associated with the ease of control of a prosthesis. Secondly, there is a need to clearly identify the extent to which studying prosthesis control with anatomically intact subjects is a valid approach.

In a study investigating novel prosthesis control approaches [8], Cipriani showed that "acceptability [of a given control scheme] is more dependent on the required attention than on the success in grasping" and urged researchers to focus on the development of prostheses that enable increased functionality, without increased attentional effort. In his paper, Cipriani does not explicitly define what he means by attentional effort, and although the focus appears to be visual attention, he used subjective feedback as his way of measuring effort [8]. Attentional effort is indeed difficult to quantify objectively, however, under normal viewing conditions it is generally accepted that the location of visual attention corresponds with the direction of gaze [12]. This close correspondence affords a valuable tool to measure visuomotor control, which in turn may provide designers with tools with which to assess the likely acceptability of new prosthesis designs.

Visual attention refers to the preferential processing of some aspect of the visual world (e.g. a location or object in a visual scene). Focused visual attention has been compared to a spotlight or zoom-lens [13,14] that shifts between relevant details of visual scenes, and is usually accompanied by a saccadic eye-movement in order to bring details into foveal vision (the central vision that is characterised by the highest acuity). Consequently, gaze-tracking has provided insights into the allocation of visual attention during reaching to grasp [15] in addition to more complex tasks such as making a cup of tea [16] or hand-washing [17]). A consistent finding from such studies is that gaze is directed to the target of movement, rather than to the hand.

These findings also extend to studies of motor learning, which broadly suggest that as tasks become well-learnt, gaze patterns shift from following the movement of a hand or tool, to looking ahead to the target of that movement [18]. For example, in a study comparing expert and naive users of a laparoscope [19], Law et al. found that experts tended to fixate and maintain gaze at the target throughout the reaching movement while novices varied in their strategies, with some using gaze to pursue the tool to the target. The differential patterns of gaze presumably reflect the need for different information during different stages of motor learning [20].

Recently, a study by Bouwsema was the first to report on visuomotor behaviours in upper limb prosthesis users [21]. This study quantified the level of skill in myoelectric prosthesis users through exploring the relationship between the clinical outcomes and different visuomotor indices. In this study, six experienced trans-radial amputees were required to perform reach to grasp and manipulation tasks with four objects (each object consisted of 2 identical-sized metal plates, separated by springs of differing stiffness. Participants were required to perform each grasp of an object using either a direct, or indirect approach. During each task, performance was evaluated based on analysis of gaze behaviour, joint angle, aperture trajectories and object compression force during manipulation. For comparison purposes, subjects also performed the Southampton Hand Assessment Procedure (SHAP) [9]. The study characterised gaze behaviour using a simple coding scheme in which the scene, recorded by a head-mounted camera, was divided into a number of categorical areas (hand, object, object and hand, endpoint and other). The authors reported time spent focusing on each of the areas in the scene and number of fixations per trial. The authors reported that all subjects focused gaze on the object being grasped for the majority of the task time, irrespective of their performance on the SHAP test. Two subjects also tended to flick back and forth between the object and the hand during task performance. This study was the first to show that the gaze patterns of users of myoelectric prostheses differ markedly from those seen in anatomically intact subjects.

The patterns of gaze during task performance are promising outcome measures for the designer, as it may reflect the strategies adopted by amputees to compensate for the absence of proprioceptive feedback and uncertainty/delays in the control system, factors believed to be central to the difficulty in using prostheses [22]. However, although Bouwsema and colleagues [21] showed distinct differences in gaze behaviours of experienced amputee users of myoelectric prostheses as compared to behaviours reported in studies of anatomically intact subjects, their analysis was limited in scope. Specifically, the object used was not one commonly encountered in everyday life. Secondly, the tasks studied were relatively simple, and thus may not be reflective of the complex multi-stage upper limb tasks commonly encountered in everyday life. Additionally, the authors only considered which objects subjects were fixated on and for how long, without considering the important aspect of gaze sequence and number of fixation transitions. Most importantly, although Bouwsema and colleagues published a series of studies, involving either amputee subjects [21,23,24], or anatomically intact subjects learning to use a prosthesis [25,26], the task sets used in the various studies differed, making comparison between performance of amputee subjects and anatomically intact subjects using a prosthesis difficult. Furthermore, none of these studies reported on the changes in gaze behaviour with learning to use a prosthesis.

Hence, it is the objective of this study to build on the existing work and assess effects of introduction and prolonged use of a myoelectric prosthesis on various aspects of gaze (i.e. gaze fixation sequence, fixation transitions, fixation duration). Our design has been motivated by the evidence that gaze is preferentially directed at the target of movement during well-learnt actions. We predict that difficulty controlling the prosthesis will be associated with longer fixation on the prosthesis itself, with skilled use marked by increasing fixation at target objects. In addition to gaze behaviour, we report the corresponding findings in conjunction with task movement time which has been shown to reflect the degree of learning [27].

## Methods

### Ethics and recruitment

The study was approved by the University of Salford Research Ethics committee (Ref # REPN09/174) and NHS National Research Ethics Service (Ref # 11/NW/0060). Seven anatomically intact individuals (four males and three females; age mean ±1standard deviation (SD): 36 ± 10 years; age range: 26-48 years) and four users of myoelectric prostheses (3 males and 1 female; age mean ±1 SD: 49 ± 10 years; age range: 35-56 years; years since myoelectric prosthesis prescription: mean ±1 SD: 20 ± 13 years, range: 2-32 years) agreed to participate in the study and gave informed consent. Of the anatomically intact individuals, six subjects were right handed and one subject was left handed. All four of the myoelectric prosthesis users were right side affected, and for three of them (S1, S2, S4) the prosthesis replaced their original dominant hand. Three subjects (S1-S3) used an Otto Bock Sensor Hand Speed and S4 used an RSL Steeper MultiControl Plus hand. S2 and S4 were fitted with a powered wrist rotator. All subjects used a two-site two-state control strategy. All subjects were able to complete upper limb functional tasks comfortably without glasses or contact lenses. All data were collected in the Movement Science Laboratory at the University of Salford, Salford, Greater Manchester, UK and the Disablement Services Centre, Manchester, UK.

### Experimental visuomotor sessions (V)

**Figure 1:**
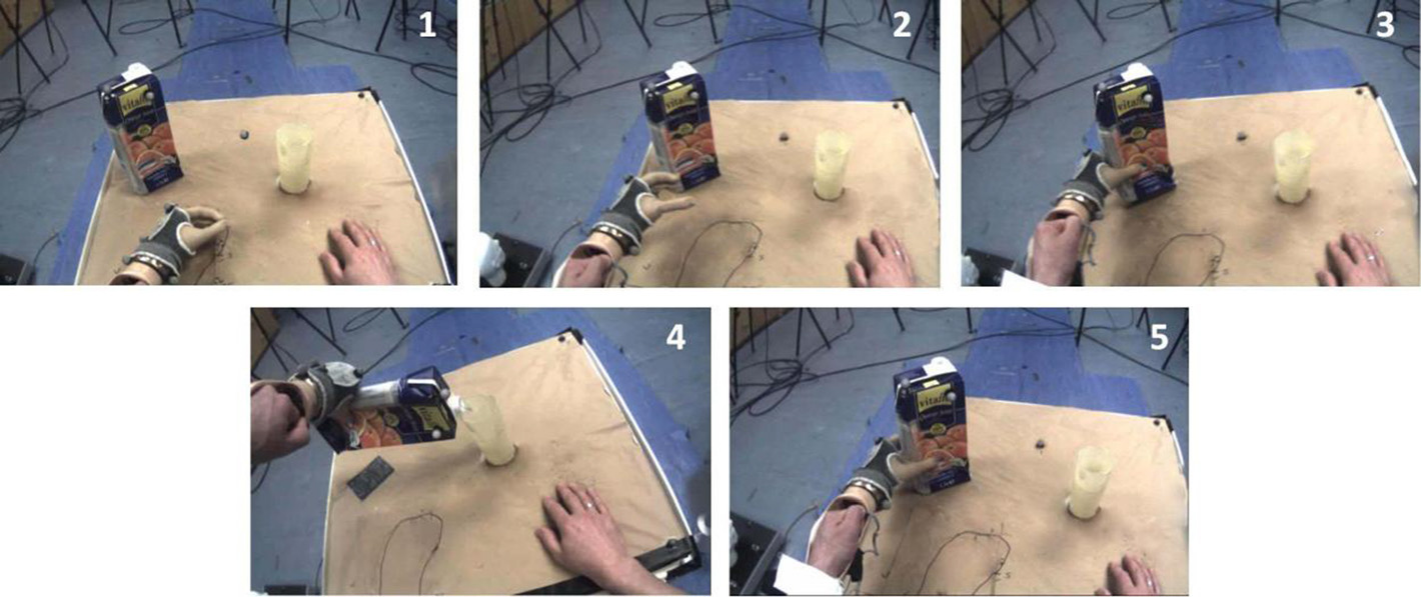
Screen shots of the carton pouring task, performed with the prosthesis simulator.

We chose to study a single multi-stage real world task (hereafter referred to as the ‘carton pouring task’, or CPT). The task involved subjects reaching with their prosthesis for the carton, picking it up, then pouring all of the water from it into a glass. Finally, the subject placed the carton back at its marked starting point, releasing the carton and returning the hand to its marked starting point (Figure 1). The task was adapted from one of the tasks in the Southampton Hand Assessment Procedure (SHAP) [9]. SHAP comprises completion of 26 self-timed tasks (12 abstract object tasks and 14 activities of daily living (ADLs)) and is a validated clinical measure of hand function. The selected carton pouring task is a functional everyday task that requires accurate movement performance and encourages attentional engagement, since it has a cost (water spillage) associated with poor performance.

The subject was seated on a chair with his/her back resting against the chair’s back and with their midline of the torso approximately aligned with the midline of the table. The upper arms were at the side of the body, elbows in a 90° flexed position, and both hands resting comfortably on the table top. The location of the hands when resting on the table was marked on paper before the start of data collection to ensure a similar arm posture and hand location at the start and end of each trial and throughout the series of repeated sessions discussed below. The carton was placed within a comfortable reach from the left hand’s start point, such that the subject was not required to lean to perform the task (the carton oriented with its posterior wall rotated 60° clockwise relative to the proximal border of the table to allow for easy grasping at minimal occurrence of occlusions of finger markers tracked with 3D cameras).

Prior to starting each attempt at the task, the subject was instructed to focus on a marked "gaze reference point" (GRP) in the centre of the table (approximately 10 cm from the distal edge of the table) to prevent subjects from fixating the carton prior to task onset. Only then was the subject instructed to begin the task. During task performance, subjects were allowed to move their eyes freely. Furthermore, head movements during task performance were unconstrained. At the end of each trial, subjects were instructed to return their gaze to the GRP. Subjects were instructed to repeat the task 12 times in each session and the first 10 trials which showed good quality data were used for analysis.

### Data collection

#### Equipment

**Figure 2:**
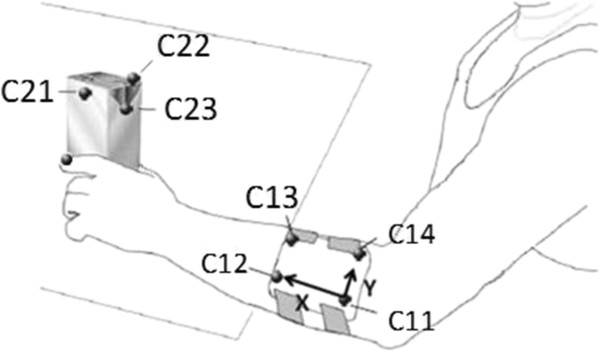
**Placement of infrared-light reflective markers whose movements were tracked during task performance.** (Note - marker F1, mounted on the thumb, is not shown in the figure).

Gaze data were captured using a head mounted iView X™ HED 2 eye-tracking system (SenseMotoric Instruments GmbH, Tellow, Germany). Kinematics were calculated from 3D reflective marker position data that were collected at 100 Hz using a ten camera Vicon 612® motion capture system (Vicon Motion Systems, Los Angles, USA). For the latter, a cluster of 4 markers (C11-C14) was used to track the movements of the forearm and another one with three markers (C21-C23) to track the movements of the carton (Figure 2). Markers were also attached to the tip of the index finger and thumb (F1, F2).

#### Data set 1

**Figure 3:**
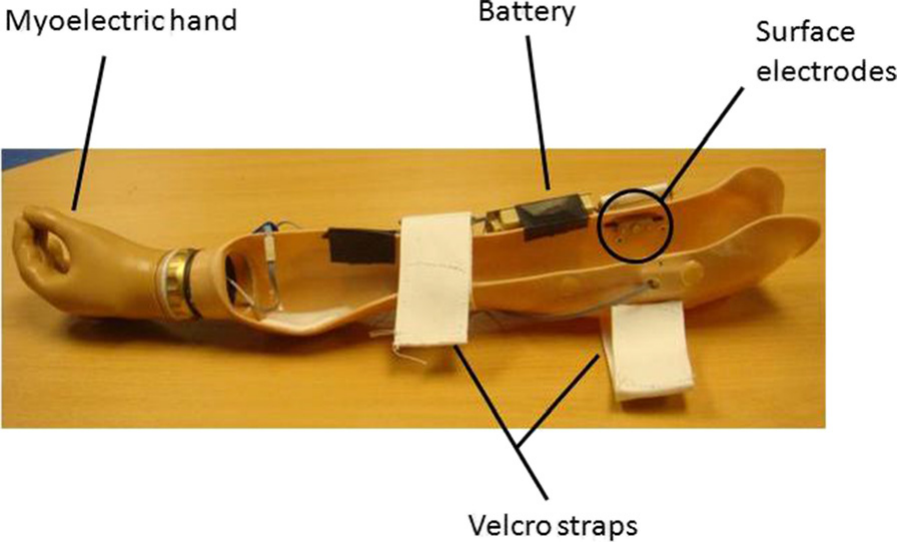
**Myoelectric prosthesis simulator.** A prosthetic socket which could be fitted over the anatomic arm was produced for every subject. The socket was equipped with a single degree of freedom left sided electrical hand (RSLSteeper "Select" Myo Electric hand (size 3 ¼")), whose opening and closing at a constant speed was controlled via EMG signals from 2 socket-located electrodes.

The purpose of Data Set 1 was to assess effects of introduction and prolonged use of a myoelectric prosthesis simulator on performance measures. Specifically, gaze behaviour and task movement time were investigated at baseline (intact anatomic hand), immediately after introduction of a myoelectric prosthesis simulator, and after repeated training sessions with the prosthesis simulator. Hence, this part of the study reflected a repeated measures design and allowed for assessment of gaze and task movement time outcome measures in relation to learning. Anatomically intact individuals were recruited for this part of the study. After familiarisation with the experimental procedure, subjects’ normal gaze behaviour during the performance of the CPT was evaluated in a single visuomotor performance session (V1) which formed the baseline for task performance with the intact anatomic hand (Table 1). As discussed above, they were next fitted with the myoelectric prosthesis simulator (Figure 3) and were then evaluated with the prosthesis simulator three times; once immediately on receiving the simulator (V2), approximately a week and then 2 weeks after initial fitting (V3 and V4 respectively). Additionally, subjects received five further separate clinical sessions, each lasting approximately 45 minutes, in which they performed the SHAP: once with the anatomic hand after V1 (SHAP1) and four times with the prosthesis simulator (SHAP2-SHAP5) as shown in Table 1. All SHAP sessions took place over approximately 2 weeks (max 14 days, min 10 days), the maximum time between successive SHAP sessions was 2 days, and the minimum was 1 day. The SHAP is a hand function test to measure in the Function and Activity domains [28]. It uses a form board and 26 self-timed tasks. It employs six abstract shapes (in two masses) and 14 simulated ADLs. Each of the 26 tasks is classified within one of the six prehensile patterns task is rated according to the time taken relative to a group of unimpaired subjects [9]. The overall score is out of 100 and is based on a weighted sum of individual grip scores, the weights depending on the frequency of use of the different grips employed by the unimpaired population. The normal population's dominant side scores above 95 [29]. The clinical evaluation sessions were performed on different days to the visuomotor performance sessions, to avoid fatigue. In addition to serving as a training tool for subjects to practice a range of tasks using the prosthesis, performing SHAP also provided a measure of hand function throughout the time course of the study to which gaze and task movement time could be compared. Subjects did not practice with the prosthesis simulator outside the SHAP sessions.

**Table 1:** Study protocol (Data Set 1): sequence of experimental assessment and training sessions

	**Condition**	**Session**
0	**Anatomic hand**	V1: Kinematics & gaze behaviour
		SHAP1
	**Prosthesis simulator**	V2: Kinematics & gaze behaviour
		SHAP2 – training
		SHAP3 – training
		V3: Kinematics & gaze behaviour
		SHAP4 – training
		SHAP5 – training
2 weeks		V4: Kinematics & gaze behaviour

#### Data set 2

The purpose of Data Set 2 was to assess gaze behaviour, task movement time and performance on the SHAP test of actual myoelectric prosthesis users in a one-shot experimental case-study; i.e. myoelectric prosthesis users were assessed in a single session, with the aim to compare their performance in relation to Session V4 of Data Set 1 (performance of anatomically intact subjects with a prosthesis simulator after repeated training to use the simulator). This was done to establish confidence in the findings that were based on Data Set 1, i.e. to provide the first evidence that use of anatomically intact subjects with a prosthesis simulator is a reasonable approach to investigate visuomotor behaviours.

### Data analysis

#### Gaze data

**Figure 4:**
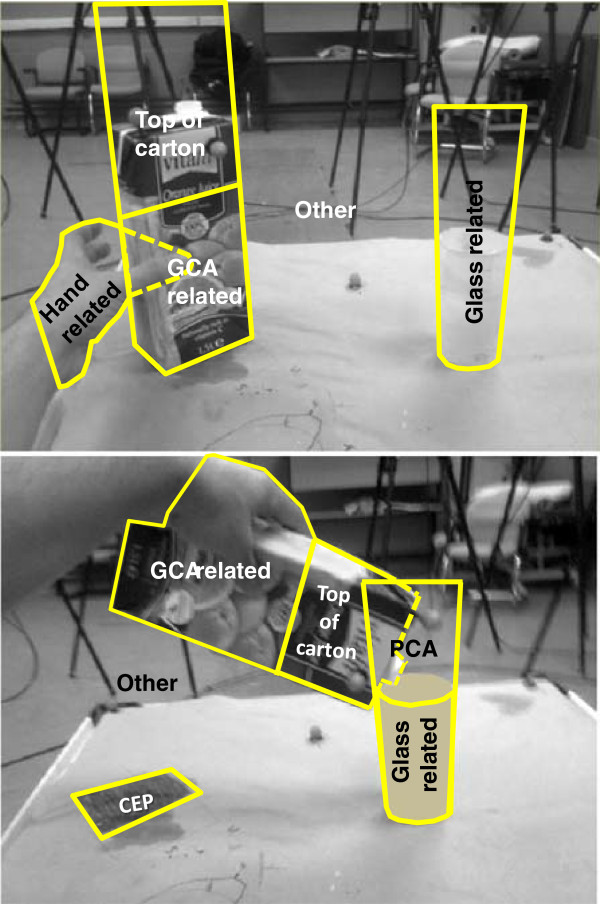
**Definition of Areas of Interest (AOIs) during reaching (top) and object manipulation phase (bottom).** GCA: grasp-critical area; CEP: carton end position. Note: after the carton was grasped, fixations on area of the hand that coincided with area of GCA were labelled as GCA related areas (not hand related areas).

BeGaze analysis software (BeGaze™ 2.3, SenseMotoric Instruments GmbH, Tellow, Germany) was used to discriminate non-fixation events (including saccades, blinks and missing data) from fixation periods. During fixation periods the software produces a red cursor indicating the point of regard (PoR) projected into the scene video, allowing for subsequent frame-by-frame analysis. At each frame, the PoR was categorised as lying in one Area of Interest (AOI), as defined in Figure 4 (blinks, saccades and missing data were all labelled as "Missing data" (MD) and further details on the coding scheme are available at in Additional file 1. To present the gaze sequence, gaze data were first divided into reaching and manipulation phases. The onset of the reaching phase was defined from the video by the onset of the hand movement; the end of the reaching phase/start of manipulation phase was defined by when the carton is seen to first leave the table, and the end of manipulation phase was defined as the point in time when the hand first releases the carton after task completion. Results were normalised by dividing each fixation period by the phase duration. Then gaze sequence was presented in stacked bars in which each coloured portion corresponds to the percentage of fixation at a single AOI. Total gaze duration at any given AOI was calculated by summing relevant fixation periods over the phase duration. Similarly, number of gaze transitions between AOIs were likewise obtained for the reaching and manipulation phase, separately.

#### Calculation of movement duration

Movement durations for reaching towards the carton and for manipulation of the carton were calculated separately. The calculation of movement times involved tracking of the movement of the forearm, index finger and thumb, as well as the position of the object. Specifically, the position data of four markers on the forearm were used for subsequent calculation of simulated accelerometer trajectories, using our previously reported approach [30]. The key events in the task were calculated based on data filtered using a 4th order Butterworth filter with a cut-off frequency of 6 Hz, followed by a 20 point centred moving average filter. Onset of movement was defined as the point in time when the X component of the accelerations measured at the centre of the forearm cluster (C11-C14) (see Figure 4) changed by 0.18 m/s^2^ relative to its resting mean value. The end of the reaching phase was defined by the onset of lifting of the carton, defined to be the point in time when the vertical position of the centre of a cluster of 3 markers on the top of the carton (C21-C23) in global coordinates exceeded a value of 10 mm above its resting location. The end of the manipulation phase was defined as the point at which the hand aperture opening velocity (rate of change of distance between the index finger and thumb markers) exceeded 0.05 m/s and the vertical position (in the global reference frame) of the carton marker cluster centre dropped below 10 mm above its original resting value. The obtained discrete time points of these "events" then allowed for calculation of phase duration (reaching and manipulation).

The onset and termination of reaching and manipulation phase for each trial were then used to calculate task duration (defined as the sum of reaching and manipulation times) and phase duration. Group means and standard deviations (SD) of phase and task durations for Data Set 1 (anatomically intact subjects) were calculated for each session, and used for statistical analysis. Means and standard deviations were also calculated for data from the amputee subjects collected at the single experimental session (Data Set 2). Due to small subject numbers, descriptive statistics are used for comparison of Data Set 1 and Data Set 2.

## Results

### Effects of introduction and prolonged use of a myoelectric prosthesis simulator on performance measures in anatomically intact subjects (Data Set 1)

#### Gaze

**Figure 5:**
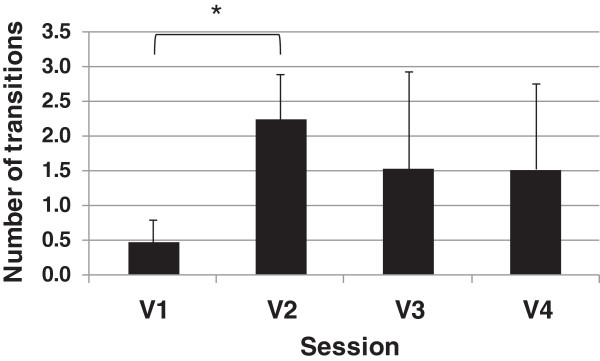
**Group means of number of transitions between AOIs during reaching phase across visuomotor performance sessions.** The asterisk indicates p < .05 between the two sessions labelled by a square bracket.

**Figure 6:**
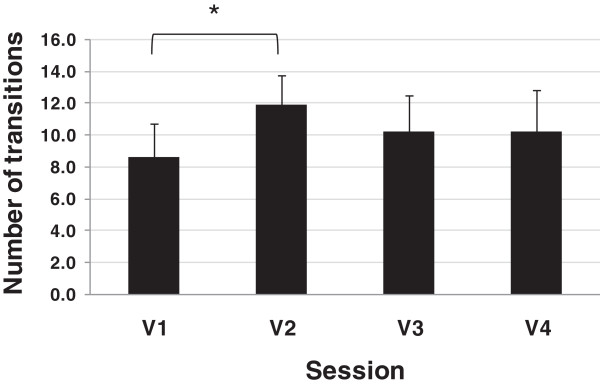
**Group means of number of transitions between AOIs during manipulation phase across visuomotor performance sessions.** The asterisk indicates p < .05 between the two sessions labelled by a square bracket.

Gaze sequencing data for all subjects across the four visuomotor performance sessions are shown for reaching and manipulation in Additional file 2. The group means of the number of transitions between AOIs during both the reaching and manipulation phases across visuomotor performance sessions are illustrated in Figures 5 and 6, respectively. In general, subjects made fewer transitions when they used their anatomic arm to perform the task. In addition, fewer transitions were required after the training period (V4) as compared to before training (V2). Statistical analysis showed a main effect of session on number of transitions in reaching (F (2, 12) = 4.22, p < .05) and in manipulation (F (2, 12) = 9.81, p < .05). When comparing pairs, only introducing the prosthesis (V1 vs. V2) significantly affected the number of transitions in reaching ((F (1, 6) = 25.14, p < .05) and manipulation (F (1, 6) = 20.70, p < .05).

As Additional file 2 illustrates, during anatomic hand use, approaching the carton (during the first half of reaching phase) was completed while fixating the gaze at the carton. During the second half of the reaching phase, when the hand is approaching and grasping the carton, gaze fixation was generally on the Top of carton AOI. In a number of trials, the end of reach phase was associated with fixation at Glass related AOI.

During prosthesis simulator use, fixation at the hand was observed notably in the first half of the reaching phase. The second half of reaching phase was predominantly associated with fixations at the GCA related AOI and occasionally at Hand related AOI. Fixation at Top of carton AOI was rarely observed; and if such fixations occurred, they were generally interrupted with fixation(s) at GCA related/Hand related. Fixation at Glass related AOI was very rarely seen at the end of reaching phase.

During the manipulation phase, fixation at GCA related AOI was observed only when subjects were using the prosthesis simulator. This was observed at the very early stage of manipulation phase and more frequently at the end of the manipulation phase.

**Figure 7:**
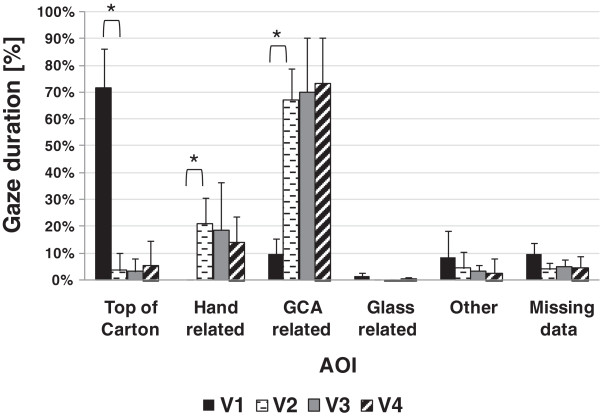
**Group means of % gaze duration at AOIs during reaching phase across visuomotor performance sessions.** The asterisk indicates p < .05 between the two sessions labelled by a square bracket.

**Figure 8:**
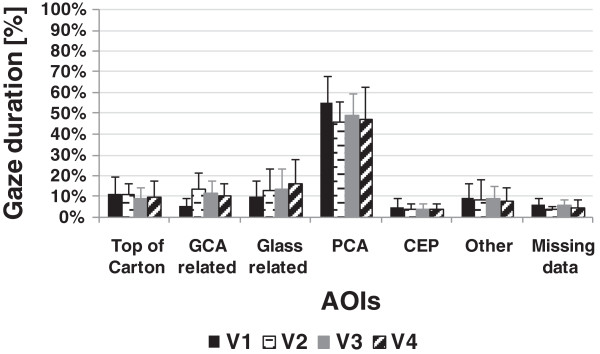
**Group means of % gaze duration at AOIs during manipulation phase across visuomotor performance sessions.** p < .05 was not found for comparison between any of the sessions.

Figures 7 and 8 show the group means of normalised gaze duration at each AOI across visuomotor performance sessions for the reaching phase and manipulation phase, respectively. During reaching, subjects focused extensively on their hand and the areas critical to grasping the carton when using the prosthesis simulator, whilst they focused on areas above their hand when using their anatomic arm. During manipulation subjects focused largely on the area critical to successful pouring, regardless of whether they used their anatomic hand or the prosthesis simulator.

#### Movement time

**Figure 9:**
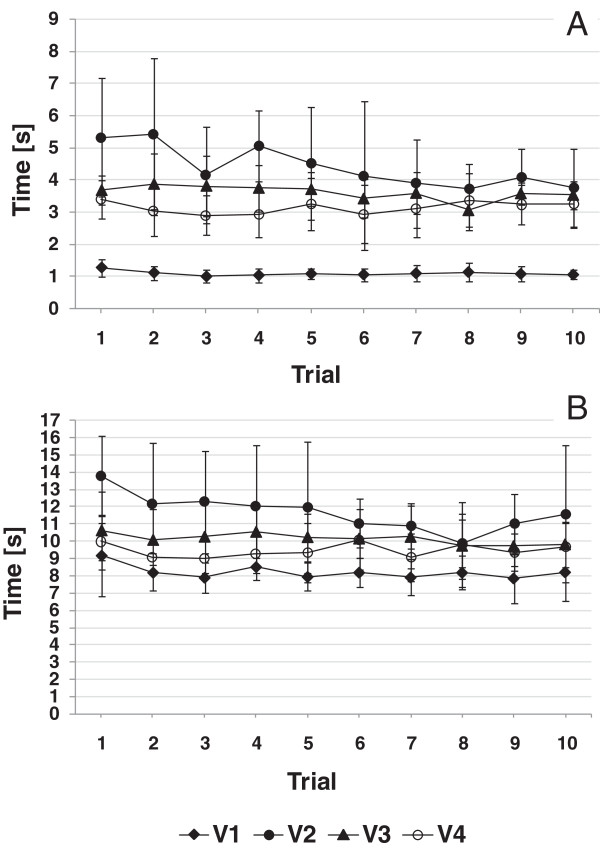
Within-session, group means of the reaching phase duration (A) and the manipulation phase duration (B).

As can be seen from Figure 9, the time taken from reach to grasp increased from just over 1 second to 5 seconds when the prosthesis was first introduced (V2). The grasping phase showed rapid reductions in time within V2, with smaller reductions between V2-V3 and V3-V4. Similar, although less distinct, patterns were seen in the duration of the manipulation phase.

Repeated Measures ANOVA showed a main effect of session on movement time in both the reaching (F (1.05, 9.42) = 189.83, p < .05) and manipulation phases (F (1.09, 9.79) = 84.94, p < .05). When comparing pairs, both introducing the prosthesis simulator (V1 vs. V2) (F (1, 9) = 286.47, p < .05) and training (V2 vs. V4) (F (1, 9) = 33.34, p < .05) were found to significantly affect the movement time in the reaching phase. Planned comparison also showed a significant effect of introducing the prosthesis simulator (V1 vs. V2) (F (1, 6) = 25.14, p < .05) and training (V2 vs. V4) (F (1, 6) = 162.47, p < .05) on movement time in the manipulation phase (F (1, 6) = 37.99, p < .05).

#### SHAP

Table 2 shows the mean (±SD) SHAP Index of Functionality (IoF) of all subjects over the study period. An increasing SHAP IoF indicates improvement in task performance. SHAP index first declined dramatically from 94 in the baseline session (anatomic hand) to 36.8 upon introduction of the prosthesis simulator. However, repeated performance of SHAP with the prosthesis simulator resulted in mean SHAP index increasing to 67.4. Repeated measures ANOVA showed a significant main effect of SHAP sessions, (*F* (2, 12) = 283.35, p < .05). Planned comparison showed a significant decrease in SHAP index when the prosthesis was introduced (*F* (1, 6) = 422.02, p < .05) and significant increase with practice (*F* (1, 6) = 258.47, p < .05).

**Table 2:** Group means (±1 group SD) of the SHAP Index of Functionality throughout the study period

	**SHAP Scores during training**				
	Session 1*	**Session 2**^**†**^	**Session 3**^**†**^	**Session 4**^**†**^	**Session 5**^**†**^
**SHAP Index of Functionality (IoF)**	94 (1)	36.8 (6.7)	51 (3.3)	60 (6.4)	67.4 (4.5)

*Anatomic hand.

^†^Prosthesis simulator.

#### Performance comparison of anatomically intact prosthesis simulator users versus amputee users of prostheses (Data Set 2)

**Figure 10:**
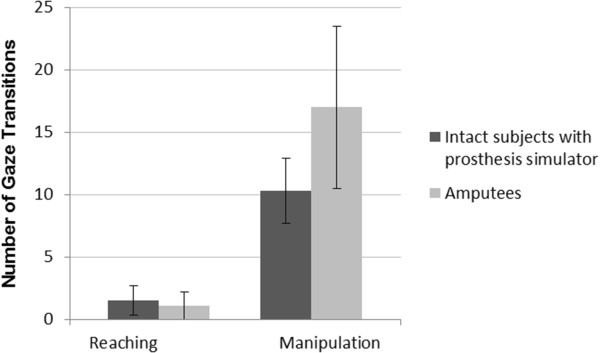
**The number of gaze transitions for intact subjects at (V4) and for prosthesis users.** Group means (SD denoted by error bars) of the number of gaze transitions for intact subjects using the prosthesis simulator at (V4) and amputees with a fitted myoelectric prosthesis during reaching and manipulation.

**Figure 11:**
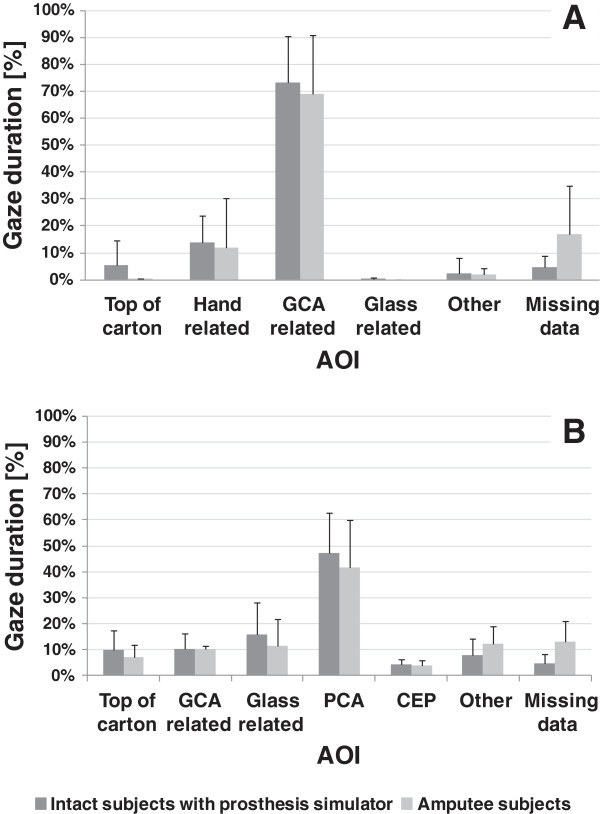
**Group means of % gaze duration at AOIs for intact subjects at (V4) and for prosthesis users.** Group means (SD denoted by error bars) of % gaze duration at AOIs for intact subjects using the prosthesis simulator at (V4) and amputees with a fitted myoelectric prosthesis during the reaching phase **(A)** and the manipulation phase **(B)**.

Gaze sequencing data during reaching and manipulation of the four amputees are shown in Additional file 2 from which the number of gaze fixation and fixation duration at AOIs were calculated. The corresponding number of gaze fixation transitions and gaze durations for intact subjects using the prosthesis simulator at the end of their training and long-term myoelectric prosthesis users are shown in Figures 10 and 11 respectively, with similar results for use of the prosthesis simulator and use of an actual prosthesis during reach, although a higher number of transitions for the amputees during manipulation*.*

Group mean durations (SD) for reaching and manipulation, for Data Set 1 (V4) and Data Set 2 are given in Table 3. Again, somewhat similar results are evident from the 2 groups.

**Table 3:** The phase durations

	**Intact subjects with prosthesis simulator (V4)**	**Amputees with fitted prosthesis**
Reaching	3.1 (0.6)	2.8 (1.2)
Manipulation	9.5 (1.1)	10.5 (1.1)

Group means (±1 group SD) of the phase durations for intact subjects using the prosthesis simulator and users fitted with a myoelectric prosthesis.

Similarly, the SHAP functionality index for intact subjects using the prosthesis simulator in V4 also agreed well with that of the amputees with their fitted myoelectric prosthesis (67.4 ± 4.5 for intact subjects with the simulator as compared to 57.5 ± 5.8 for the amputees with their prosthesis).

## Discussion

### Gaze behaviour

When performing a familiar upper limb task gaze usually follows a particular characteristic routine path involving fixation at certain key AOIs, and thus the number of transitions between AOIs is normally low. In contrast, for difficult and/or novel tasks, gaze behaviour tends to be erratic, with more frequent transitions between AOIs [19,31]. With practice, the number of transitions is reduced and the search strategy becomes more consistent. Our results agreed with the general patterns reported in the literature. Generally, the graphs shown in Additional file 2 show that the anatomic hand reaching was almost fully executed while fixating at the carton (mainly at Top of the carton followed and less often at GCA related AOI), therefore showed few transitions. There were over four times as many transitions between AOIs in V2 compared to V1 in the reaching phase, although less clear differences were seen in the manipulation phase (Figures 5 and 6). Over the course of practice the number of transitions was lower at both V3 and V4, compared to V2, for both reaching and manipulation but changes were not significant.

In line with previous research [16], during reaching with the anatomic hand, subjects did not generally focus on hand related areas, or the grasp critical area (GCA) (Figure 7). Instead, subjects tended to fixate their gaze at the areas which may be of relevance to the subsequent action ("look-ahead fixations" [32]), notably the Top of Carton area, and a very small amount of time focusing on glass related areas, which may indicate planning for subsequent parts of the task (see also gaze sequences in Additional file 2). In stark contrast, at V2 prosthetic reaching was mostly associated with attention to the hand related (particularly during the first half of reaching phase) and GCA areas (Figure 7 and Additional file 2). Attention given towards the hand related area is probably associated with concern regarding the hand configuration and location and suggests the use of visual feedback to guide the hand and/or ensure hand opening, while approaching the carton. The attention given to the GCA (particularly during the second half of reaching phase) may indicate both planning the reach and guiding the hand-carton interaction. Attention to all these areas largely precluded the subjects from planning ahead for the manipulation phase.

With practice, from V2-V4, the duration of the fixation at the GCA related during reaching increased slightly, probably as a result of a shorter fixation on the hand area (during the first half of the reaching phase). This might indicate that the prosthesis simulator users begin to develop the ability to plan the movement trajectory of the prosthesis simulator towards the carton. It can be assumed however that, even with training, grasp formation still relied to an extent on visual feedback being gathered during the action, as gaze fixation(s) in the second half of the reaching phase was mainly at the GCA related AOI.

Much smaller differences were seen in data from the manipulation phase (Figure 7 and Additional file 2). As mentioned earlier fixation at GCA related AOI during the manipulation phase was observed (in some trials) only during prosthesis simulator use. Fixation at GCA related AOI was notably in the stage in which carton slippage was highly possible (during the first third of the manipulation during which the carton was transported towards the glass and tilting the carton to pour water were executed). This suggests uncertainty of the hand state. In a few trials, short intervals of gaze fixation(s) at GCA related were observed within the second half of manipulation phase (while pouring the water). This may be also due to the lack of direct proprioceptive feedback from the prosthetic hand thus the user needed to visually ensure that the grasp security. Fixation at the GCA related AOI was also observed right at the end of the manipulation phase during which the simulator user was about to release the carton from the hand. Generally, the duration of fixation at GCA related AOI appeared to slightly decline with training (Figure 8). Nevertheless, as Additional file 2 indicates, releasing the carton from the prosthetic hand continued to be largely associated with fixation at GCA related AOI. Therefore, releasing the carton from the prosthetic hand (as in grasping) may have required visual attention (this observation may not generalise to other, for example, rigid objects).

When comparing results from V4 with gaze data from the study of four amputees (Figure 10), there is reasonable agreement in the number of gaze transitions in the reaching phase, but less so in the manipulation phase; perhaps reflecting the familiarity of the anatomically intact subjects by V4 with, what may to the amputees be an unfamiliar unilateral task, pouring water from a carton.

The comparison of the patterns of gaze durations observed at V4 with the anatomically intact subjects and gaze duration data from amputee subjects shows a reasonably good agreement between the two patterns of data. Both data sets reflecting a clear focus on GCA related areas for around 70% of the reaching duration, with approximately 15% of the time spent focusing on hand related areas. Again, rather similar patterns were observed in the manipulation phase data between V4 and the amputee data sets. The rather different choice of task and coding scheme makes comparison with the findings of Bouwsema et al. [21] difficult. However, their study also showed that amputees focus gaze on the hand, a behaviour almost never seen in studies of anatomically intact reaching and grasping [16].

### SHAP and movement time

The SHAP IoF scores at V1 were 94, just under the normal range (95-100) [33], dropping on first use of the prosthesis simulator to 36.8. With practice, at SHAP5, anatomically intact subjects reached a mean IoF of 67.4, reflecting a rapid learning effect. It is interesting to note that recent work with a prosthesis simulator has also shown a practice effect [34] and the improvements between V2 and V4 may reflect true skill acquisition, familiarity with the test, or a combination of the two. Unsurprisingly, the duration of the reaching phase also followed a similar pattern, with average reaching duration going from around 1 second at V1 to 4.4 seconds at V2, then dropping to 3.1 seconds by V4. Of particular interest is the apparent rapid reduction in duration over just the first 10 attempts with the prosthesis simulator. Again, the manipulation phase showed less clear effects.

The amputee subjects in our study had a mean IoF 57.5 ± 5.8, slightly lower than for the anatomically intact subjects with the simulator at SHAP5. On average, our amputee subjects performed slightly better and were more consistent in their IoF scores than the subjects recruited to the Bouwsema study [21]. The duration of reaching and manipulation phases were similar between the two groups (i.e. anatomically intact vs. amputee subjects) (Table 3).

### Study limitations

Although we found significant differences in gaze behaviours between the prosthesis users and those using their anatomical hand to perform the carton pouring task, further work is needed to understand how these finding may generalise to other upper limb tasks. Additionally, the broad similarities in the visuomotor behaviours of the anatomically intact subjects with prosthesis simulators and amputee subjects need to be treated with caution due to the age difference between the two groups.

## Conclusions

This study is the first to report on the visuomotor behaviours seen in subjects using a myoelectric prosthesis to perform a multi-stage real world task. The results from the study of intact subjects (Data Set 1) clearly show the major influence of prosthesis introduction on gaze behaviours, particularly in the case of this task, in the reach to grasp and releasing actions. Generally, the observed gaze behaviours indicate that when using the prosthesis simulator, subjects were poor at using gaze to plan subsequent actions in the task, maybe due in certain parts of the task to uncertainty in grasp security. The gaze behaviours were surprisingly insensitive to practice, and encouragingly, we saw similar gaze behaviours in the four amputee subjects we studied (Data Set 2).

Also, as expected, subjects showed a dramatic increase in the time from reach to grasp on first use of the prosthesis simulator. The practice effect was dramatic in the first session (V2), suggesting subjects were very quickly finding better ways of controlling hand opening. Again, the effects were seen most clearly in the reach to grasp analysis, rather than in the analysis of the manipulation phase data. There were similarities in the performance of anatomically intact subjects at V4/SHAP5 with the amputee subjects.

It is possible to speculate that the gaze strategies adopted may be influenced by the design of the prosthesis; as artificial proprioception provides the user with more information on the state of the prosthesis, so we would expect gaze behaviours to return to patterns which are characteristic of anatomically intact subjects. The findings encourage more work in this area to provide designers with appropriate tools with which to evaluate emerging upper limb prostheses. Further work studying anatomically intact subjects with prosthesis simulators would also be of benefit, to clarify when such an approach is suitable.

## Abbreviations

ADLs: Activities of daily living; ANOVA: Analysis of variance; AOI: Areas of Interest; CPT: Carton pouring task; EMG: Electromyographic; GCA: Grasping Critical Area; GRP: Gaze reference point; IoF: Index of Functionality; MD: Missing data; PCA: Pouring Critical Area; PoR: Point of regard; SD: Standard deviations; SHAP: The Southampton Hand Assessment Procedure.

## Competing interests

The authors declare that they have no competing interests.

## Authors’ contributions

Study concept and design: MS, LK, AG, ST and PK. Subject recruitment: MS, JM and JK. Acquisition of data: MS and JM. Analysis and interpretation of data: MS, LK, AG and ST. Drafting of manuscript: LK, MS and ST. Critical revision for important intellectual content: LK, AG, ST and PK. Study supervision: LK, AG and ST. All authors read and approved the final manuscript.

## Supplementary Material

Additional file 1 Gaze coding scheme. Click here for file

Additional file 2 Gaze sequence. Click here for file
