# Supplementary material for: Visuomotor behaviours when using a myoelectric prosthesis
Source: J Neuroeng Rehabil. 2014 Apr 23;11:72. doi: 10.1186/1743-0003-11-72 (PMC4022381; doi:10.1186/1743-0003-11-72)
Supplement: Additional file 1 — Gaze coding scheme. [file 1743-0003-11-72-S1.pdf]

Supplement 1: Gaze coding scheme

The coding scheme presented in this paper is a simplified version of a previously developed coding scheme, which included a larger number of areas of interest (AOI) (http://usir.salford.ac.uk/29401/). The simplification process involved amalgamation of adjacent AOIs, in cases where the functional relevance of the combined AOIs was retained.

Each AOI has a unique functional relevance to the task performance. The functional relevance of some AOIs is phase-dependant, and indeed, some AOIs only exist in a single phase. The table below provides a detailed description of AOIs.

**AOIs**	**Definition**	**Example**	**Phase**
Hand related	The area that the hand occupies (up to ulnar and radial styloids, or equivalent on the prosthesis wrist) in addition to the area confined between the thumb and the index finger. This AOI also exists when the point of regard is close to the boundary of the hand and moving with the hand.	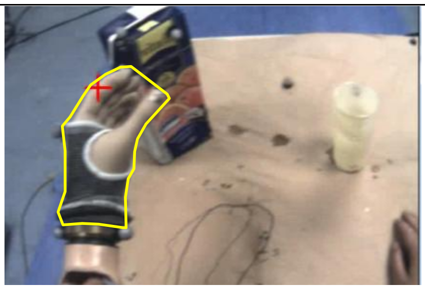	Reaching phase only.

Grasping critical area (GCA) related	**Reaching phase:**<br>The area on the carton that is located below the level of the index finger when the hand first grips the carton. The AOI is defined as follows:<br>1. At a frame when the hand is fully grasping the carton, draw a line between the highest visible point of the index finger and the lateral border of the carton, parallel to the thumb.<br>2. GCA related is the area on the carton that is below this line.<br>**Manipulation phase:**<br>As defined in reaching phase in addition to the area occupied by hand related AOI.	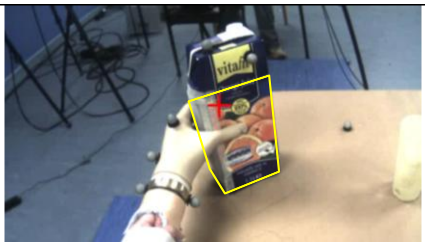<br>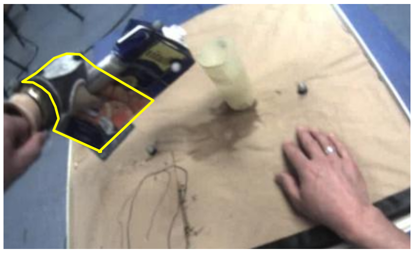	Reaching and manipulation phase.

Top of carton	**Reaching phase:** The area on the carton above the upper border of the GCA related in addition to the area located directly above the carton (up to the edge of the captured scene). Also this AOI exists when the point of regard is close to the boundary of the carton and moving with the hand. **Manipulation phase:** As defined in reaching phase. Note: When Top of carton AOI overlaps with Glass related AOI, the overlapped part of Top of the carton is defined as PCA (see example).	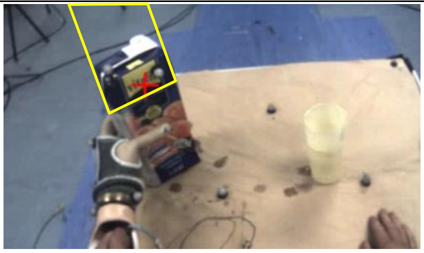 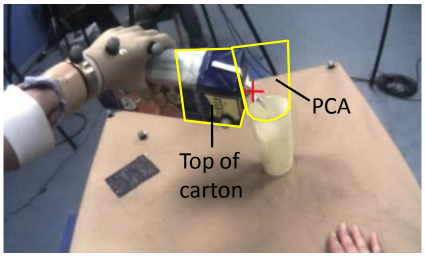	Reaching and manipulation phase.
Glass related	**Reaching phase:** The area that the glass occupies (this includes the rear half of brim and any inner part of the glass), in addition to the	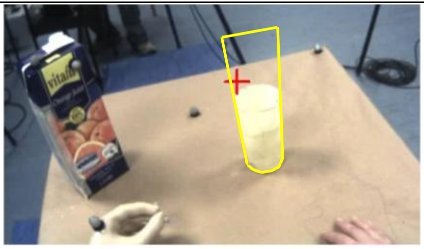	Reaching and manipulation phase.

	area located directly above the glass which is defined as follows:<br>1. The upper boundary is defined by a line running approximately parallel to the table, starting at the highest point on the carton when it intersects with the area above the glass, during pouring.<br>2. The two sides are defined by lines extending from each edge of the glass.<br>**<u>Manipulation phase:</u>**<br>As defined in reaching phase.<br>Note: Only the outer part of the glass (excluding the rear half of brim and any inner part of the glass) defines as Glass related AOI when PCA AOI comes to exist (see example).	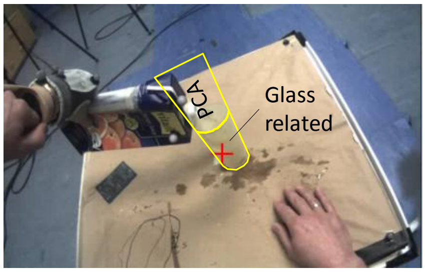	

Pouring critical area (PCA)	An AOI emerges when Top of carton AOI overlaps with Glass related AOI. PCA occupies a part of Glass related AOI; the part that locates directly above the glass (including the rear half of brim and any inner part of the glass).	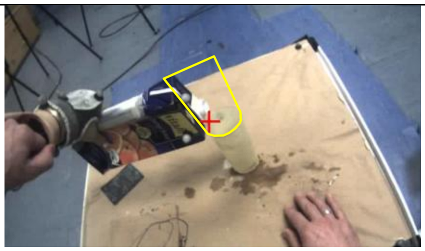	In manipulation phase only.
Carton end-point (CEP)	The area on the table at which the carton is placed.	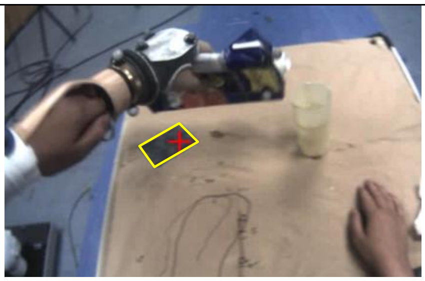	In manipulation phase only.
Other	**Reaching phase:**<br>The area that does not belong to any of the other AOIs.<br>**Manipulation phase:**<br>As defined in reaching phase.	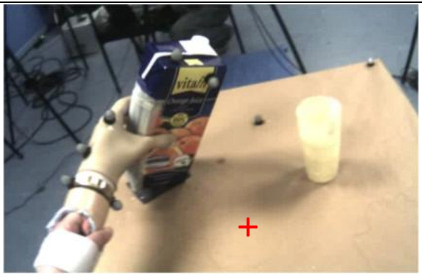	In reaching and manipulation phase.
Missing Data (MD)	**Reaching phase:**<br>When the gaze indicator disappears.	N/A	In reaching and manipulation phase.

	**<u>Manipulation phase:</u>**<br><br>As defined in reaching phase.		
