# Supplementary material for: Visuomotor behaviours when using a myoelectric prosthesis
Source: J Neuroeng Rehabil. 2014 Apr 23;11:72. doi: 10.1186/1743-0003-11-72 (PMC4022381; doi:10.1186/1743-0003-11-72)
Supplement: Additional file 2 — Gaze sequence. [file 1743-0003-11-72-S2.doc]

**Supplement 2: Gaze sequence**

- 1. **Data Set 1: Anatomically intact subjects** **using the prosthesis simulator (pages 1-9).**

The graphs below show the observed gaze sequence in 7 anatomically intact subjects during the completion of carton pouring task. In each graph below, the trial number is represented on the vertical axis. The horizontal axis represents the task duration normalised to 100%. The gaze fixation sequence in a given trial is presented in a stacked bar in which each coloured segment denotes a gaze fixation at a particular AOI, the length of each coloured segment corresponds to the duration of the fixation at the AOI. The first 28 graphs listed below show the observed gaze sequence during the reaching phase while the use of the anatomical (graphs labelled V1) and while the use of the prosthetic hand (graphs labelled V2, V3 and V4) for each subject separately. The following 28 graphs show the gaze sequence during the manipulation phase while using the anatomical and prosthetic hand for each subject separately.

- - 1. **Reaching phase**

- - 1. **Manipulation phase**

- 1. **Data Set 2: Amputee users using a prosthesis (10-11)**

The graphs below show the Gaze sequence in 4 trans-radial amputee subjects during the completion of carton pouring task using their prosthesis. In each graph below, the trial number is represented on the vertical axis. The horizontal axis represents the task duration normalised to 100%. The gaze fixation sequence in a given trial is presented in a stacked bar in which each coloured segment denotes a gaze fixation at a particular AOI, the length of each coloured segment corresponds to the duration of the fixation at the AOI. The first 4 graphs show the gaze sequence during the reaching phase for each subject separately. The following 4 graphs show the gaze sequence of 10 trials during the manipulation phase for each subject separately.

- - 1. **Reaching phase**

- - 1. **Manipulation phase**
